# Raman-Activated, Interactive Sorting of Isotope-Labeled Bacteria

**DOI:** 10.3390/s24144503

**Published:** 2024-07-11

**Authors:** Sepehr Razi, Nicolae Tarcea, Thomas Henkel, Ramya Ravikumar, Aikaterini Pistiki, Annette Wagenhaus, Sophie Girnus, Martin Taubert, Kirsten Küsel, Petra Rösch, Jürgen Popp

**Affiliations:** 1 Leibniz-Institute of Photonic Technology, Member of the Leibniz Research Alliance—Leibniz Health Technologies, 07745 Jena, Germany; sepehr.razi@leibniz-ipht.de (S.R.); nicolae.tarcea@uni-jena.de (N.T.); thomas.henkel@leibniz-ipht.de (T.H.); aikaterini.pistiki@leibniz-ipht.de (A.P.); 2 Cluster of Excellence Balance of the Microverse, Friedrich Schiller University Jena, 07743 Jena, Germany; martin.taubert@uni-jena.de (M.T.); kirsten.kuesel@uni-jena.de (K.K.); 3 Institute of Physical Chemistry and Abbe Center of Photonics, Friedrich Schiller University, 07743 Jena, Germany; ramya.motganhalli.ravikumar@uni-jena.de (R.R.); petra.roesch@uni-jena.de (P.R.); 4 Aquatic Geomicrobiology, Institute of Biodiversity, Friedrich Schiller University Jena, 07743 Jena, Germany

**Keywords:** Raman spectroscopy, microfluidic sorting, isotope labeling, bacteria

## Abstract

Due to its high spatial resolution, Raman microspectroscopy allows for the analysis of single microbial cells. Since Raman spectroscopy analyzes the whole cell content, this method is phenotypic and can therefore be used to evaluate cellular changes. In particular, labeling with stable isotopes (SIPs) enables the versatile use and observation of different metabolic states in microbes. Nevertheless, static measurements can only analyze the present situation and do not allow for further downstream evaluations. Therefore, a combination of Raman analysis and cell sorting is necessary to provide the possibility for further research on selected bacteria in a sample. Here, a new microfluidic approach for Raman-activated continuous-flow sorting of bacteria using an optical setup for image-based particle sorting with synchronous acquisition and analysis of Raman spectra for making the sorting decision is demonstrated, showing that active cells can be successfully sorted by means of this microfluidic chip.

## 1. Introduction

Microbes, such as bacteria and archaea, from environmental habitats such as soil or aquifers are hard to isolate or even cultivate in laboratory conditions [1,2,3,4,5,6]. Nevertheless, gaining further information, especially information on the microbial activity in such habitats, can give initial insights into such microbial communities [7,8,9,10,11]. Here, the possibility to detect microbial activity with a non-destructive and non-interfering method would be useful. One way to distinguish bacteria according to their metabolic properties is to label them with isotopes before applying Raman experiments [12,13].

Raman spectroscopy in combination with a microscope allows for measuring single bacterial cells and analyzing the entire content of the cells [14,15,16]. In addition, Raman microscopy does not alter or destroy the bacteria [17]. Metabolic labeling of bacteria with stable, heavy isotopes like D, ^13^C, ^15^N, or ^18^O, so called stable isotope probing (SIP), leads to the incorporation of these isotopes into cellular biomass [18,19,20,21]. In Raman spectra, this leads to shifts in the original Raman bands to lower wavenumber positions, allowing for the detection of these incorporated isotopes according to the variation in the biomolecules’ signatures [22,23]. With this method, bacteria can, e.g., be cultivated in D_2_O, enabling their differentiation according to their general activity [9,24,25]. Other studies have revealed preferred nutrition, like carbon [26,27,28,29,30,31,32] or nitrogen sources [33,34,35]; the possibility to degrade certain substances [31]; phosphate solubilization [36,37]; metabolic pathways [9,38,39,40,41], growth rates [42]; antibiotic resistance [43]; and even monitoring of bacterial interaction [44].

To obtain further information about the active bacteria, it is helpful not only to count the abundance of bacteria within a certain sample but also to sort them according to their properties [45,46,47,48,49].

In recent years, powerful microfluidic approaches for image-activated cell sorting have been established [50]. Cell sorting plays a pivotal role in microbiology, enabling researchers to pick and separate specific individual cells from complex microbial communities for further analysis. This isolation of specific cells allows for downstream molecular analyses or cultivation efforts. Especially, combinations of Raman spectroscopy, laser trapping, and microfluidic chips have emerged as powerful tools for precise and efficient cell sorting [13,45,47,48,51,52,53,54,55,56,57,58,59,60,61,62,63,64,65,66,67,68,69]. These technologies offer unprecedented capabilities for analyzing individual microbial cells, enabling a downstream analysis of the sorted cells with different methods [39,40,46,69,70].

For sorting, different techniques are available, like dielectrophoresis [56,71,72,73,74,75,76,77], hydrodynamic focusing [47], microholes [78], laser tweezers [11,49,79,80,81,82,83,84,85], acoustofluidics approaches [86], and magnetic application [87,88]. Using laser tweezers inside microchannels allows not only a contactless analysis of the bacteria [89,90,91], but also trapping of bacterial cells in the laser spot and sorting into the corresponding outlet channels [45]. This combination of information acquisition and subsequent sorting is therefore widely used to evaluate and sort bacteria cells by means of Raman-SIPs [45,48,56,61,92,93,94].

For particle displacement, the low trapping forces of optical tweezers must overcome the Stokes viscous forces of the trapped particle relative to the surrounding fluid. This relative velocity is the sum of the velocity of the XY stage and the velocity of the liquid at the location of the trap within the sorting chamber. For RACS applications, relative fluid velocities up to 500 µm/s have been already reported [45,89], which conforms with volumetric flow rates in the range of 0.25 to 10 nL/s, dependent on the channel cross-sectional area. Particularly, when using multiple outlets for collecting different kinds of cells, these tiny flow rates must be kept stable over the entire sorting time (ten minutes to hours). In this context, long-term, stable, and controllable laminar co-flow can be used by using pressure-based fluid management in combination with throttling capillaries [95]. Such a co-flow approach can be used to create a stationary separation fluid lamella between the cell sample stream and the outlets for the sorted cells, avoiding any crosstalk.

**Figure 1:**
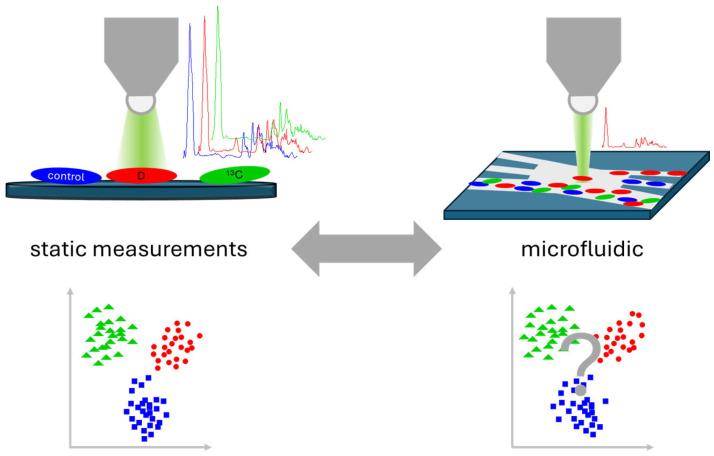
Schematic layout of the measurement strategy: comparing static reference Raman-SIPs measurements with results from Raman-SIPs for microfluidic sorting.

Here, we report on an experimental infrastructure for Raman-activated sorting of bacteria and its application to discriminate and retrieve individual bacteria based on their metabolic activity status (Figure 1). For sorting, a novel laminar co-flow-based flow cell design was developed, where helper fluid streams prevent unwanted microfluidic crosstalk between the main sample stream and the two outlets for collecting the sorted bacteria. Metabolic activity was measured based on the formation and accumulation of isotope-labeled biomass inside the cells using D- and ^13^C-enriched culture media. Here, the Raman spectra from static reference measurements were compared to those from Raman sorting. From the single-cell Raman data, two classes can be discriminated and collected into the two cell collecting reservoirs of the developed RACS system.

## 2. Materials and Methods

### 2.1. Sample Preparation

For this study, the bacterial strains *Escherichia coli* (*E. coli*) DSM 498 and *Micrococcus lylae* (*M. lylae*) DSM 20,318 purchased from the German Collection of Microorganisms and Cell Cultures (DSMZ, Braunschweig, Germany) were used. All media and chemicals were purchased by Sigma Aldrich (Munich, Germany). The bacteria were cultivated in 5 mL of M9 broth with additional glucose. The culture media were prepared either with water, 30% heavy water (D_2_O), or with ^13^C-labeled glucose. All cultures were grown with 120 rpm rotational shaking at 37 °C for 24 h. One milliliter of each culture was centrifuged for 5 min at 3500× *g* to discard the supernatant, and the pellet was washed with deionized water. For reference measurements, small droplets of the bacteria suspension were dried on Ni foil (h+s Präzisionsfolien, Pirk bei Weiden, Germany).

For the microfluidic experiment, the resulting pellets were re-suspended in 1 mL of 0.5 × TW buffer (50v% tap water (Water Hardness 3.5°DH) and 50v% deionized water) to reduce osmotic stress and diluted to a cell concentration of 10,000 cells/µL, which proved to be suitable for single cell measurements. This strategic dilution significantly minimized the potential simultaneous trapping of multiple cells via the implemented laser tweezer during sorting experiments. Washed, labeled, and non-labeled samples were then mixed together at ratios of 1:1 to be used in the sorting experiments and evaluation of the microfluidic system performance. Cell concentrations were measured using a cell counting capillary slit chamber with capillary slit height of 25 µm and benefiting from an optical microscope (Zeiss Jenatech 58092, Carl Zeiss, Jena, Germany).

### 2.2. Raman Spectroscopic Reference Measurements

The reference Raman spectra were recorded using a Raman microscope BioParticleExplorer (Rap.ID Particle Systems GmbH, Berlin, Germany) coupled with a 532 nm excitation wavelength from a frequency doubled Nd:YAG laser (LCM-S-111-NNP25; Laser-export Co., Ltd., Moscow, Russia). The laser beam was focused on the sample via a 100x air objective (MPLFLN-BD, NA = 0.90, Olympus Corporation, Tokyo, Japan) with a spot size of below 1 µm and a laser power of about 9 mW at the sample. The integration time was 10 s per single bacterial cell spectrum. The single-stage monochromator (HE 532, Horiba Jobin Yvon, Bensheim, Germany) was combined with a 920 lines/mm grating. The detector was a thermoelectrically cooled, charge-coupled device (CCD) camera (DV401-BV; Andor Technology, Belfast, Northern Ireland) with a spectral resolution of about 8 cm^−1^. On each measurement day prior to each measurement, 4-acetamidophen was measured for wavenumber calibration. To minimize cultivation artifacts, at least 4 replicates of each experiment were analyzed.

### 2.3. Raman Measurements in Combination with Microfluidics

For the microfluidic Raman measurements, the above-described Raman microscope (BioParticleExplorer, Rap.ID Particle Systems GmbH, Berlin, Germany) was modified to allow sample inspection with transmission light microscopy. A blue LED transmission light source M470D3 (470 nm, Thorlabs Inc., Newton, MA, USA) with a condenser NA of 0.3 (f = 30 mm, D = 18mm) was used for the bright field transmission mode illumination. To block longer wavelength emissions of the light source which could superpose the Raman signal, a short-pass filter FES0500 (Thorlabs Inc., Newton, MA, USA) with a cut-off wavelength of 500 nm was integrated into the parallel beam section of the light source. The microfluidic chip device was mounted onto the motorized XY stage SCANplus 75 × 50 (Märzhäuser Wetzlar GmbH & Co. KG, Wetzlar, Germany) of the system and the z-focus was achieved by a 13 mm vertical travel stage (Thorlabs Inc., Newton, MA, USA). For video image monitoring during Raman data acquisition, a monochrome camera (2.2 Megapixel) ace acA1920-155um (Basler AG, Ahrensburg, Germany) with an additional short-pass filter FES0500 was used.

A 532 nm continuous-wave diode-pumped laser (Cobolt Samba™ 150, Cobolt AB, Solna, Sweden) served dual purposes, both as a laser tweezer and for Raman excitation. Emitting output light at approximately 90 mW power, the laser was focused onto flowing cells within the microfluidic substrate using a 60x NIR-Apo Nikon water-dipping microscope objective lens, where the captured image region had a size of 203 × 128 µm^2^. A single-stage monochromator, HE 532 (Horiba Jobin Yvon, Bensheim, Germany), featuring a 920 lines/mm fixed grating was fiber-coupled to the Raman microscope. The original 50 µm core coupling fiber was replaced with a 100 µm core to relax the system confocality. The detection was conducted with a thermoelectrically cooled charge-coupled device (CCD) camera, IDUS DU420A-BV (Andor Technology, Belfast, UK), equipped with a 1024 × 255 array of 26 µm pixels and capable of thermoelectric cooling down to ~−50 °C.

### 2.4. Fluid and Sample Management

The microfluidic sorting chip with 5 fluid interconnecting holes on its top face was integrated into a microscopy slide-sized chip mount with vertically arranged PTFE tube adapters (OD 1/16″, ID 0.5 mm, length: 40 mm). One side of the adapter tube was flanged using the Easy Flange Kit (VICI AG International, Schenkon, Switzerland). Adapter tubes were pressed with the flange on the fluid ports of the chip by the fluid interconnection block of the chip mount. Silicon tube sleeves with a length of 15 mm (ROTILABO^®^ silicone tube, Standard design, ID = 1.0 mm, OD = 3.0 mm, Carl Roth GmbH, Karlsruhe, Germany) were used to connect the adapters to the tubing of the fluid management system. Afterwards, the chip mount was inserted into the sample holder of the Raman device with fluid connections pointing downwards.

For pressure-based fluid management and control, the FlowEZ-345 mbar fluid management system (Fluigent, Le Kremlin-Bicêtre, France) connected to a Fluiwell 4-channel 0.5 mL high pressure sampling reservoir bench (Fluigent, Le Kremlin-Bicêtre, France) was used. Sampling reservoirs for cell suspension in deionized water (sample fluid) and deionized water (helper fluid) were connected with the sample and helper fluid inlet of the chip using PEEK capillaries with a length of 500 mm, an outer diameter (OD) of 1/16″, and an inner diameter (ID) of 0.075 mm (PEEK Tubing, 1/16 × 0.075 mm ID, green striped, Altmann Analytik GmbH & Co. KG, Munich, Germany). Outlets were connected to the fluid collecting vials (1.5 mm PE vials with screw cap, with a 1.6 mm hole at the center of the cap) using PEEK HPLC capillaries (OD 1/16′, ID 0.1 mm, black striped). The capillary length was 250 mm for the channel 1 and channel 2 outlets and 85 mm for the waste outlet.

For the initial filling of the microfluidic system, vials with degassed 0.5 × TW buffer were attached to the respective channels of the sampling reservoir bench. Initial filling was performed with pressure settings of 300 mbar until bubble-free filling of the chip was confirmed by microscopic inspection.

For Raman-activated sorting, the vial with the cell suspension was attached to the respective channel of the sampling reservoir bench and the first pressure of 58 mbar was applied to the helper fluid, followed by increasing the pressure of the sample reservoir to 26 mbar. Penetration of sample fluid into one of the sorted outlets can be avoided by carefully maintaining the pressure of the helper fluid reservoir above the pressure level of the sample reservoir.

### 2.5. Automation and Developed Software

Most of the trapping and measurement process is controlled by custom-written software. Different measurement scenarios can be used. A standard sorting experiment requires the user to set the regions where particles are to be trapped, analyzed, and respectively released. In the trapping region, an automatic hunting of particles is performed, significantly increasing the throughput (analyzed particles/time unit) of the system. Automatic recognition if a particle is trapped is conducted both by evaluating a certain spectral region of the Raman spectra (e.g., C-H stretching vibration at ~2900 cm^−1^) or by video frame analysis (object detected at the location of the optical trap). Continuously evaluating the trapped object also helps to estimate the contamination in channel 1 by cells meant for channel 2 (lost while crossing the flow which goes to channel 1). For now, the sorting decision (either to channel 1, 2, or discarding the object trapped) is conducted by the user, with work in progress to also automate this step by having a discriminator algorithm trained.

### 2.6. Microfluidic Device Preparation

For the preparation of the microfluidic devices “PAP-B-v0.1”, an established technology workflow was applied. Microfluidic devices were built by covering a glass channel wafer (diameter 100 mm, thickness 0.7 mm, material BorofloatR33, Plan Optik AG, Elsoff, Germany) equipped with the micro channel structures and fluid interconnection holes with an unstructured cover wafer (diameter 100 mm, thickness 0.2 mm, material BorofloatR33, Plan Optik AG, Elsoff, Germany). Channel structures were etched with a depth of 70 µm into the channel wafer using a mixture of 600 g ammonium fluoride (Merck, Darmstadt, Germany) dissolved in 900 mL water and 300 mL 40% hydrofluoric acid (Merck, Darmstadt, Germany), at 70 °C for 2 h. A photo lithographically structured nickel–chromium alloy (60/40) thin film with a thickness of 60 nm was used as a mask. Fluid interconnecting holes were drilled by ultrasonic grinding using a cylindrical grinding tool with a diameter of 0.5 mm and a borcarbide abrasive with a grain size of 320 µm (Heinrich Wasels GmbH, Altena, Germany). For bonding, first a 60 nm silicon layer was deposited on the cover wafer, followed by anodic bonding with the channel wafer at 380 °C for 30 min at a voltage of 440 V. After sewing the wafer into chips (size 16 × 25 mm^2^) the silicon was removed from the internal channels by treatment with 15% w/vol sodium hydroxide in water at 50 °C for 60 min, followed by subsequent washing of the channels with deionized water.

### 2.7. Data Pre-Processing and Multivariate Data Analysis

The data pre-processing and analysis were performed using RAMANMETRIX software 5.0 (https://ramanmetrix.eu, accessed on 12 June 2024). Prior to analysis, several pre-processing steps were performed, like de-spiking [96] and wavenumber calibration, with polynomial fit function with a degree of 3. Afterwards, sample spectra were baseline corrected using a Sensitive Nonlinear Iterative Peak (SNIP) clipping algorithm with 40 iterations and vector normalization. Spectra were then truncated to the relevant range of 400–3050 cm^−1^. For the reference database, a Principal Component Analysis in combination with a Linear Discriminant Analysis (PCA-LDA) was used with 8 principal components (PCs) for N-fold cross validation, whereas for the microfluidic database, 5 PCs for N-fold cross validation were chosen [97]. The sensitivity and specificity for the three-class-model were calculated according [98].

## 3. Results and Discussion

### 3.1. Design and Function of the Microfluidics Device

**Figure 2:**
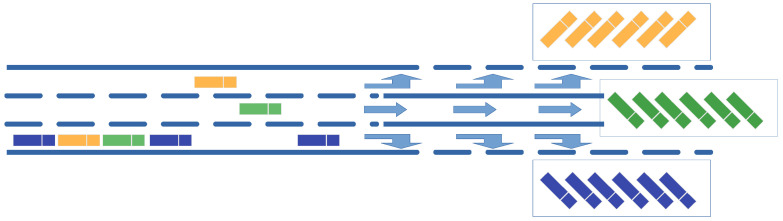
Three-lane highway connected with three cargo terminals for different goods. All trucks arrive from the rightmost lane of the highway and move to their dedicated lane in the arrangement section of the station. Finally, they leave the sorting section to the dedicated cargo terminal.

One of the major strengths of continuous flow microfluidic sorting is its ability to combine high-throughput analysis with the opportunity to select and separate target particles for subsequent analysis and processing. Many microfluidic implementations make use of the laminar co-flow of fluids in a straight sorting channel which ends in a branch for delivering the individual fluid lamella into different outlets of the chip. The principle of operation can be compared to a multi-lane highway ending in cargo terminals for collecting different types of goods, as outlined in Figure 2. As in laminar co-flow, the lanes are only virtually separated, and cargo objects may switch on demand from one lane to another one.

In order to implement the highway scenario depicted in Figure 2 in a microfluidic setting, forces need to be applied for moving the particles from the inlet lane to one of the sorted outlet lanes. Optical tweezers have proven to be very flexible and easy to handle tools for this kind of application [79,99,100].

The complexity of such an implementation increases with the number of sorted outlets due to the need for precise volume flow control in all branches of the microfluidic network. Even if a microfluidic system has only two outputs, one of them would have to be controlled in order to reduce the number of degrees of freedom of the system to one, such that the behavior of the system is precisely defined. The maximum speed at which the trapped objects can be moved relative to the surrounding liquid results from the available trapping forces of the optical trap. For *E. coli*, which has also been used in our experiments, velocities up to 80 µm/s have been experimentally confirmed using a 1064 nm laser trap operated at 30 mW [101].

However, for practicable applications, the utilized fluid transport velocity must be kept below the aforementioned value. For the system presented here, we found a maximum trap moving velocity in the range between 40 and 80 µm/s for *E. coli*. The precise management of these low-flow velocities in a microfluidic setting is challenging. Therefore, it is common, to reduce the complexity of the microfluidic system by using only a single outlet for the sorted particles and high-precision syringe pumps for fluid and sample management with sample flow rates of 1 nL/s and on-chip fluid transport velocities of 500 µm/s [45,46]. To operate our chip with two sorted outlets and one waste outlet, the volumetric flow rates at the three outlets need precise control. In contrast to the conventionally used syringe pumps, this is managed by pressure-driven fluid management and flow control. In our system, pressure-driven flow is used to move the fluids through the chip. Flow rates in the individual branches of the chip are controlled by using well-defined throttling capillaries to connect the chip with the pressurized fluid reservoirs.

For this RACS approach, we developed a new sorting chip combining microfluidic elements and the possibility to measure Raman spectra directly in the chip. This allows the collection of the sorted cells in two outlet reservoirs. At the same time, the risk of crosstalk between the cell containing sample fluid and the outlets of the sorted cells is minimized by separating these fluid streams by a fluid sheet of helper fluid, as shown in Section 3.2.

**Figure 3:**
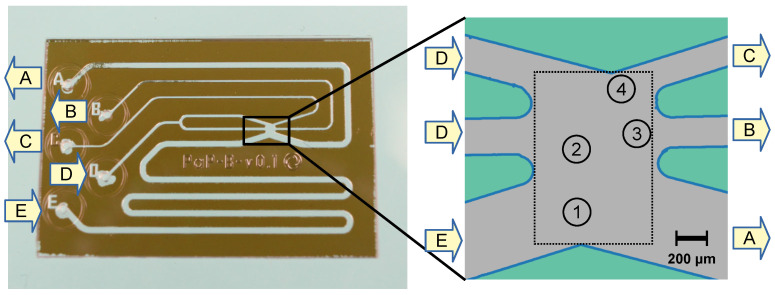
Micrograph of the prepared sorter chip (size 16 × 25 mm^2^) with the enlarged sorting chamber for Raman-activated cell sorting. A: waste; B: channel 1; C: channel 2; D: helper fluid; E: inlet of cell suspension; 1: initial laser position; 2: measuring position; 3: releasing position for channel 1; 4: releasing position for channel 2. The sorting box (rectangular region) available for moving the trapped cells has a size of 720 × 1060 µm^2^. For more details, see text.

The sorting chamber for Raman-activated sorting (Figure 3) has two fluid inlets (helper fluid (D) and sample fluid (E)) and three outlets for collecting the cells to the channel 1 (C), channel 2 (B), and waste collecting reservoirs (A). Cells enter the sorting chamber through the sample inlet (E), passing the chamber in its lower part. Here, a cell can be picked up by the laser spot (1) and moved to a defined measurement position (2), where it is permanently surrounded and washed by pure helper fluid. After measuring and analyzing the Raman spectrum in the chamber, it can be moved—based on the retrieved Raman information—to one of the sorted outlets for release (3 or 4). These two sampling outlets allow for the simultaneous collection of two different groups of bacteria at the same time without the need for re-measuring the cells. During this operation, the flow must be permanently managed in a way that cells entering the system from the sample inlet cannot float to one of the sorted outlets without being actively sorted by the optical trap. Therefore, the flow is maintained in a way that unwanted crosstalk of the sample cell suspension to one of the sorted outlets is prevented by a surplus of helper fluid flow into the chamber.

An important consideration for the microfluidic measurement configuration was to make it easily operational on existing Raman microscopes. Many of such laboratory Raman microspectroscopy devices are already equipped with a precision sample positioning (e.g., microscope table), transmission light microscopy functionality, and one or more lasers, usually with available emission power well in excess of what most biological samples are currently measured with. This opens up the possibility of using the same laser for both optical trapping and manipulating the small sample objects, as well as for the Raman measurements. However, drawbacks to this approach exist: a mismatch between the trapping position and the laser focus waist leads to suboptimal excitation of the Raman signal. This can be partially compensated by relaxing the confocality of the system’s detection path. The damage threshold of the biological sample might be a limiting factor in how much laser power can be used for trapping. A trade-off between optical tweezer strength and the type of sample used exists. Particles exhibiting a strong absorption for the given laser wavelength generally cannot be manipulated without experiencing strong thermal damage.

**Figure 4:**
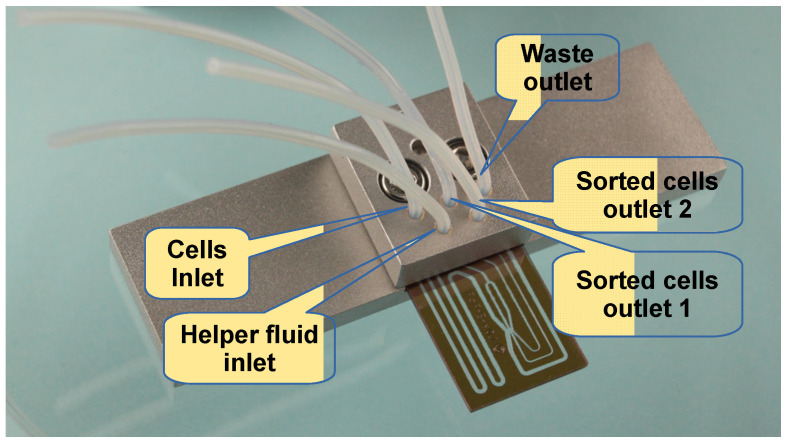
Microfluidic sorter chip, mounted into a microscopy slide-sized frame for integration into standard sample holders for microscopy. For more details, see text.

For the experiments reported here, the microfluidic chip was mounted onto the microscope table like any other microscope slide (Figure 4). This provided the interfaces for fluid interconnection as the required construction space for microscopy even for large sized immersion fluid objectives with short working distances.

Particle manipulation was performed by moving the complete microfluidic chip while the trap position stayed fixed relative to a laboratory frame. The chip was connected to the microfluidic system, and the sample and helper fluid flow were activated by changing the pressure inside the fluid reservoirs to 26 mbar for the sample and to 58 mbar for the helper fluid. This created a continuous laminar co-flow flow of the sample fluid and the helper fluid in the sorting chamber, with a near-wall particle transport velocity of about 40 µm/s.

The trapped cell is shifted to position 2 for Raman measurement, where it is flushed by the cell-free helper fluid. Raman spectra are recorded and displayed to the operator who is making the sorting decision by pressing a selection button on the control software. Now the cell is moved to the respective outlet positions 3 or 4 and released into one of the respective outlet channels (channel 2—position 3 or channel 1—position 4) by closing the laser shutter. That way up to 120 cells can be sorted per hour. This currently rather low throughput results from the transport speed of the trap of 40 µm/s and the length of the required transport path of 960 µm. Therefore, a low velocity is required to minimize the risk of losing trapped cells during transportation. For each sorting operation, about 25 s are required for moving the cell from the trap position to the sorted position. An additional 5 s in total are spent for Raman acquisition, decision making, release, and return to trap position 1. Reducing the time required for transportation must be seen as the key to increasing throughput in future implementations. A short video (Video S1) in the Supplementary Materials illustrates the suggested system performance.

### 3.2. Experimental Evaluation of the Flow Pattern

To create the required flow patterns inside the sorting chamber of the chip and check the control parameters, a prediction by microfluidic network simulations was performed. As mentioned above, the flow passing through the sorting unit of the chip must be precisely controlled in order to efficiently suppress any crosstalk from the sample transport path to the sorted outlets. This is managed by feeding a surplus of helper fluid into the chip, which prevents particles from reaching one of the sorted outlets. The microfluidic control parameters for this situation in terms of pressure settings and throttling capillary configuration can be obtained from simulations. Such software-based prediction of the behavior of complete microfluidic networks [95,102] supports the design and reliable operation of such complex microfluidic applications reported here, like sorting with multiple outputs. Such a system can be treated as a resistance network and solved by applying the Kirchhoff rules [103] to determine the required length and inner diameter of the throttling capillaries.

**Figure 5:**
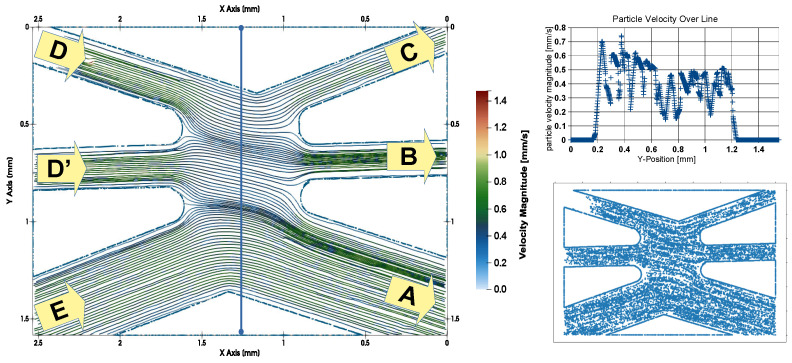
The left part shows the measured fluid trajectories in the sorting chamber for optimized flow rate settings with inflow at the left- and outflow at the right-hand side of the sorting chamber (A: waste; B: channel 1; C: channel 2; D, D’: helper fluid; E: inlet of cell suspension). The upper-right part shows the velocity profile along the blue line at the left. Measuring and zero flow supporting points used in flow field reconstruction are displayed in the lower-right part.

Particle Tracking Velocimetry (PTV) [104] has been selected as the method for measuring the flow fields inside the chamber. For data acquisition, polystyrene tracer particles with a diameter of 2.2 µm were added to a density-matched fluid (ST105 Buffer) with a density of 1.05 g/mL at a concentration of 10,000 beads/µL. This particle suspension was used for all inlets. The flow was started by pressurizing the fluid reservoirs, and image sequences of the flow cell were recorded using a 5× magnifying microscope objective. Particle motion was tracked, and the velocity at various particle locations was extracted from the PTV data analysis results. This yields an unstructured dataset of measurement points from which the fluid velocity at any point can be obtained by interpolation. Additional boundary points with zero fluid velocity were added to the dataset at wall positions to implement a zero-flow wall boundary condition. The same was applied to the edges of the image. All measured positions, including the added zero velocity boundary points, are shown in Figure 5, lower right.

For flow field interpolation, a triangulated 2D mesh was created by Delaunay triangulation [105]. The interpolated data were used for calculating the streamlines. For triangulation and streamline generation, the paraview toolkit [106] was applied without smoothing the data.

Helper fluid entered the sorting chamber from the positions D and D’ (see Figure 5, left). The particle suspension for sorting arrived from E. The pressure settings were designed in a way that ensured the particle transport velocity magnitude is nearly the same in all three inflow channels: D, D’, and E. This is confirmed by the streamlined representation within the inflow section of channels D, D’, and E. Without active sorting, all particles entering the sorting chamber from E should completely leave the chamber through outlet A. Therefore, a surplus of helper fluid is applied, which leaves the chamber through outlet A for unsorted particles. This forms a separation fluid sheet which prevents an inflow of particle-loaded sample fluid from inlet E to one of the sorted outlets, B or C. The fluid velocities in the center of the sorting chamber are analyzed in the upper-right part of Figure 5. The velocity in the upper part (low y position values) of the chamber is slightly increased compared to the lower part. Additionally, we see strong velocity fluctuations along the line. These are caused by the PTV algorithm. In contrast to the classical averaging approach used in cross-correlation-based particle imaging velocimetry [107], the PTV algorithm calculates the correct velocities for each observed particle without any averaging. Since the particles pass the capillary slit chamber at different z positions within the parabolic fluid velocity profile, the observed strong variations in the velocity magnitude of the individual tracer particles reflect their different z positions in the capillary slit chamber. At the same time, the directional variation in the flow is smooth since the flow direction is uniform over the height of the capillary slit channel, which explains the superior quality of the streamline representation derived from the experimental data.

### 3.3. Raman Reference Database

To evaluate the sorting capabilities of the above-described microfluidic approach, bacteria with different size ranges and morphologies were used. As representative species, the Gram-negative *E. coli* and Gram-positive *M. lylae* were chosen. *E. coli* are rods in the size range of 1–1.5 µm diameter and 2–4 µm in length. *M. lylae* are cocci with a diameter of approximately 0.5–0.8 µm. For all measurements, only single bacterial cells were used, without differentiating between them.

**Figure 6:**
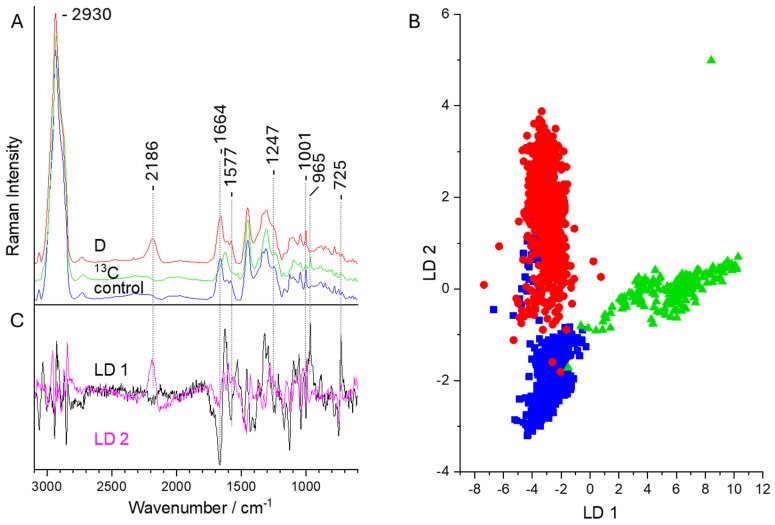
(**A**) Raman mean spectra of single bacteria (*E. coli* and *M. lylae*) without isotope labeling (control, blue) and with labeling with D (red) or ^13^C (green). (**B**) LD plot for the discrimination of different labeled bacteria with the subsequent loading vectors (**C**).

First, the bacteria were characterized without the microfluidic approach as reference measurements to demonstrate the D or ^13^C labeling. In Figure 6A, the Raman mean spectra for the reference database containing bacteria (*E. coli* and *M. lylae*) without isotope labeling (control) and with isotope labeling are shown. These Raman data were measured with an optimized laboratory Raman microscope, ensuring the maximum S/N ratio. Since Raman spectroscopy on bacteria is a phenotypic method [11], the normal Raman spectra of unlabeled cells exhibit Raman bands which can be assigned to all biochemical components of the cells. Proteins contribute to the amide I signal at 1664 cm^−1^, the amide III contribution at 1247 cm^−1^, and the phenylalanine (Phe) ring breathing vibration at 1001 cm^−1^. DNA gives rise to the band at 1577 cm^−1^, which can be assigned to the C=N stretching vibrations of the purine bases and the ring breathing mode of adenine at 725 cm^−1^. Furthermore, a strong Raman signal around 2930 cm^−1^ due to the C-H stretching vibration can be found in bacterial Raman spectra.

In the D-labeled bacteria, several Raman signals are red shifted. The most prominent example is the occurrence of the C-D stretching vibration at 2186 cm^−1^ in the so-called wavenumber silent region, where usually no other bacterial Raman signals appear [12].

In the case of ^13^C labeling, the changes in the Raman spectra are not this obvious as for H/D exchange, since the mass variation from ^12^C to ^13^C is not so dramatic compared to the change from ^1^H to D. Here, the most obvious change can be found for the position of the amide I vibration. This vibration, which is attributed to the C=O stretching vibrations of the amide bonds, is shifted from 1664 to 1625 cm^−1^. In addition, the C–N stretching vibration and the N–H bending mode of the amide III signal shifts to 1235 cm^−1^, and the Phe band shifts to 965 cm^−1^. This huge shift in the ring breathing vibration of Phe from 1001 to 965 cm^−1^ can be explained by a benzene ring completely labeled with ^13^C [108,109].

For the reference database, Raman spectra of the bacteria without isotope labeling and with isotope labeling were collected. With these Raman spectra, a PCA-LDA database was established, showing the good separation between unlabeled and D- or ^13^C-labeled cells (Figure 6B). The classes of the control bacteria (blue) slightly overlap with the deuterium-labeled cells (red). The reason for this lies in the partially low signal-to-noise ratio of the *M. lylae* when labeled with D. The LD loadings in Figure 6C reveal the differences in the Raman signals, which are mainly due to the C-D stretching vibration for D labeling (LD 2) and the amide I and Phe signals for ^13^C labeling (LD 1). The classification of the reference database achieves an accuracy of 90.4%.

Since *M. lylae* obviously do not incorporate D as easily as *E. coli*, time-dependent measurements were performed. To evaluate how many cells do incorporate isotopes during cultivation, the bacteria were cultivated for 1 or 2 days and measured directly after washing (Table 1). Since *E. coli* grows faster than *M. lylae* in the M9/glucose medium, almost all bacteria were already isotopic labeled after 1 day. For *M. lylae*, even after 2 days the incorporation ratio was much lower, with 72% of the investigated cells. This finding correlates with the aforementioned overlapping signals in Figure 6B.

Since transportation between the microfluidic setup and the reference setup can be an issue in experimental work, a possible storage time was checked to ensure that this would not alter the isotope concentration within the bacteria. Therefore, the temporal changes in isotope labeling (Table 2) were evaluated for *E. coli*. Here, we cultivated *E. coli* in M9-D_2_O and analyzed 120 cells by means of Raman spectroscopy. In all bacterial cells, the C-D stretching vibration could be observed. The prepared bacteria in deionized water were then stored at 4 °C. After washing all cells, a subsequent measurement after 24 h storage resulted in 100% of the bacterial cells with a C-D stretching vibration band. After 4 d storage, 97% of the cells still showed a C-D Raman signal. This result shows that it takes some time before the cells lose the D labeling and it is possible to store labeled samples for some time.

### 3.4. Combining Raman Measurement and Microfluidics

**Figure 7:**
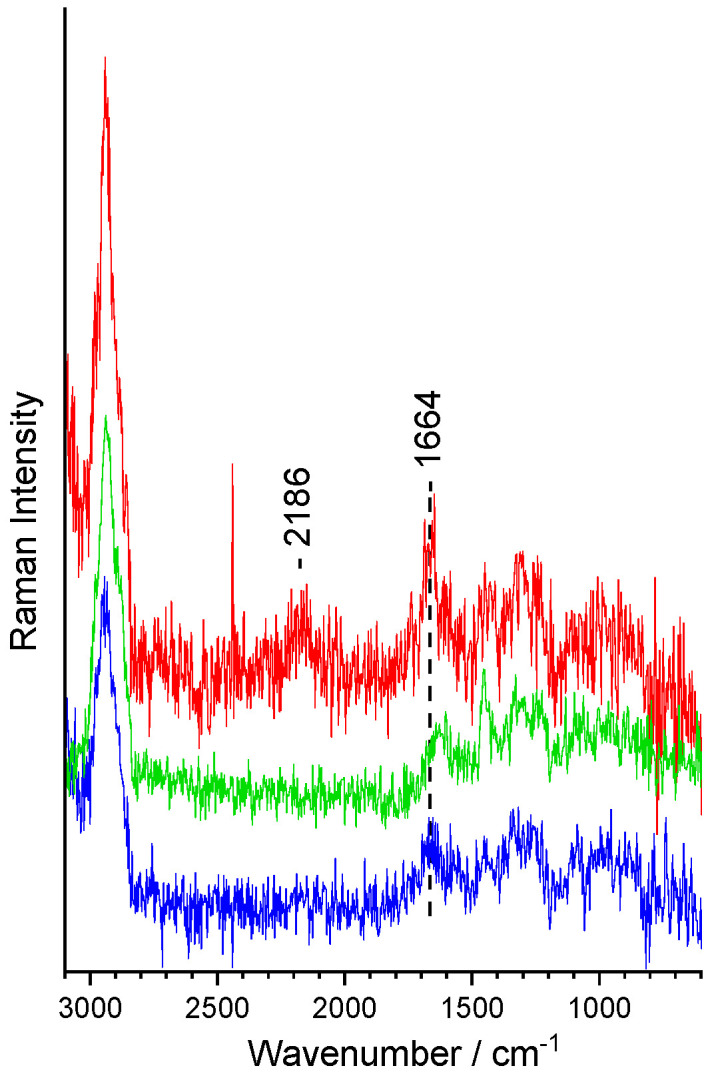
Unprocessed Raman spectra of single bacteria (*E. coli*) without isotope labeling (control, blue) and with labeling with D (red) or ^13^C (green) measured in the microfluidic channel.

We used the Raman excitation laser to record Raman spectra and at the same time as an optical trap for transporting the cells to the corresponding outlet. Figure 7 exemplarily shows the raw Raman spectra of trapped cells. For the microfluidic experiments, only *E. coli* cells were used since here, the isotope incorporation was more stable and reliable, as reported in Table 1. Since the bacteria are directly measured inside a microfluidic channel, the water content is much higher as compared to the reference data of dried bacteria shown in Figure 6. In addition, the S/N ratio is also lower, and the background is higher due to the presence of the microfluidic channel and the chip material in proximity to the channel. Nevertheless, the characteristic C-D stretching vibration can be clearly recognized and used to distinguish between D- (red), ^13^C-labeled (green), and unlabeled cells (blue) (see Figure 7). In addition, the shift in the ^13^C signal can also be observed in the raw data. No morphological changes in the *E. coli* cells in the microfluidic channel before and after measurement were observed. In addition, the Raman spectra of the single cells do not exhibit any sign of photodamage [110,111,112,113,114,115], and it was even possible to afterwards grow the sorted bacteria on Petri dishes.

**Figure 8:**
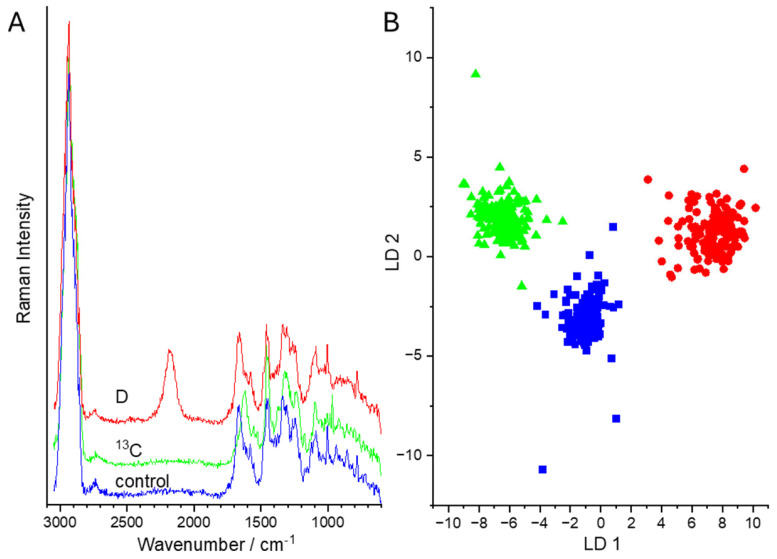
(**A**) Raman mean spectra of bacteria (*E. coli*) during sorting without isotope labeling (control, blue) and labeling with D (red) or ^13^C (green) measured in the microfluidic channel. (**B**) LD plot for the discrimination of labeled and unlabeled bacteria.

In Figure 8A, the Raman mean spectra of bacteria without isotope labeling (control, blue) and with labeling with D (red) or ^13^C (green) measured inside the microfluidic channel are displayed. Here, we can see that all spectral features can be observed. However, the S/N ratio is still lower as compared to the reference spectra shown in Figure 6. Using this spectral information inside the microfluidic channel results in a discrimination value of 99.8%. The corresponding scatterplot is shown in Figure 8B. Here, the tree classes of control (blue), and D-labeled cells (red) and ^13^C-labeled bacteria (green) can also be easily distinguished.

During sorting, the Raman spectra of the two sorting channels were collected and evaluated against the established microfluidic database of Figure 8, with the three classes of non-labelled (control), ^13^C-labeled, and D-labeled *E. coli*. With this database, we could assign the Raman spectra of the sorted bacteria exemplarily for two experiments (Table 3): In the first experiment, non-labeled cells were differentiated from D-labeled *E. coli*. The validation achieved an accuracy of 91.5% to the respective classes. Misclassification arose for control Raman spectra when the S/N ratio was still too low and the Raman spectra were classified as ^13^C-labeled. In addition, D-labeled cells were classified as control when the amount of D in the cells was not high enough to give rise to a prominent C-D signal. For the second experiment, ^13^C-labeled and D-labeled *E. coli* were used. Here, all ^13^C-labeled bacteria were identified correctly, but for D-labeled *E. coli* some spectra were falsely identified as control or ^13^C-labeled bacteria, resulting in an overall balanced accuracy of 95.8%.

The sorting process itself is prone to faults since the operator’s expertise is used for the sorting decision. Especially for C-D stretching vibrations with a low S/N ratio, this decision is challenging using the raw spectra of Figure 7. Therefore, in such situations, the intuitive selection into one channel or the other can be demanding. Here, an automated sorting algorithm based on a previously established database would not only increase the sorting accuracy but would also be more stable for longer measuring campaigns.

Nevertheless, the results presented demonstrate the power of the designed microfluidic approach for RACS in terms of sorting bacteria Raman spectra in different classes for further downstream analysis.

## 4. Conclusions

In our work, we designed a novel optofluidic infrastructure for the Raman-activated cell sorting of isotope-labeled bacteria that supports the separation and recovery of two different isotope-labeled bacterial cells. The reliability of the sorting process benefits from the implemented microfluidic device, where the laminar co-flow of sample and auxiliary fluids was utilized to efficiently suppress crosstalk between the sample cell stream and the outlets dedicated to the collection of the actively sorted cells. The microfluidic design allows one to sort two differently labeled cell moieties simultaneously, and to control all the volumetric flow rates more precisely in a continuous and uninterrupted flow. Currently, the sorting decision is left to the operator, which limits the application to visually distinguishable spectra.

At the same time, computer models for software-assisted discrimination based on the Raman signatures of individual cells were developed and used for software-assisted revalidation of the operator’s sorting decisions, and for the final evaluation of the purity of the cell populations obtained by sorting. The throughput was confirmed at 120 sorting operations per hour. Possibilities for increasing the throughput arise from improving the holding forces of the optical trap in conjunction with shortening the transport paths in the sorting chamber.

The manual decision necessary for sorting the bacterium cells in their respective channels is susceptible to failures due to the noisy raw spectra, but this will increase with longer measuring times. To minimize the operator bias, the next step will be to implement an automated sorting decision which should enable the sorting of a higher number of cells with a higher precision over a longer time period.

Overall, the established sorting chip now paves the way for to distinguish, e.g., between active or non-active bacteria, autotroph and heterotroph species, or between any other classes in environmental or patient samples which can be distinguished by means of Raman spectroscopy by sorting two (metabolic) states at a time.

## Figures and Tables

**Table 1:** Number of bacterial cells exhibiting the isotope signal after washing.

	Total	With C-D	Without C-D	With ^13^C	Without ^13^C
*E. coli*					
After 1d	101	98	3		
	60			60	0
After 2d	121	121			
*M. lylae*					
After 2d	176	127	49		

**Table 2:** Number of bacterial cells after washing and storage in H_2_O at 4 °C exhibiting the isotope signal.

*E. coli*	Total	With C-D	Without C-D
After washing	120	120	0
After 1d	120	120	0
After 4d	120	117	3

**Table 3:** Validation of the sorting experiment.

Predicted True	^13^C	Control	D	Sensitivity/%	Specificity/%
Sorting control vs. D					
Control	3	130	0	97.7	85.4
D	0	23	134	85.4	100
Sorting ^13^C vs. D					
^13^C	112	0	0	100	98.5
D	2	9	120	91.6	100

## Data Availability

The raw data supporting the conclusions of this article will be made available by the authors on request.

## Supplementary Materials

The following supporting information can be downloaded at: https://www.mdpi.com/article/10.3390/s24144503/s1, Video S1: title: Raman-activated, interactive sorting of isotope labeled bacteria.
